# Dr. Scott, on Adhesion of the Placenta

**Published:** 1800-05

**Authors:** Walter Scott

**Affiliations:** Surgeon, Northumberland Militia Bury


### *To the Editors of the Medical and Phyfical Journal.*

Gentlemen,

In reading your valuable publication, the Medical and Phyfical Journal, for March, I obferve Mr. Peck's opinion, "On the expediency of the early delivery of the placenta." An idea of the fame kind occurred to me fourteen years ago; if you deem the following cafe of morbid adhefion of the placenta to the fundus uteri, &c. worthy of your attention, the inferting of it will confer a favour upon,

Gentlemen,

Your obedient fervant,

WALTER SCOTT, M. D.

*Bury, April* 5, 1800. Surgeon, Northumberland Militia

---

ON the 6th of May, 1787, I was called upon to attend R. Robfon, of Crugden, in the county of Northumberland, about thirteen miles from Stamfordham, a ftout, healthy, middle-fized woman, of an atrabilious temperament, and in or about the fortieth year of her age; exceedingly well made, and in her fecond labour. The particulars of the cafe were, fo far as I could learn from the midwife: That fhe had been called upon the day before; that R. Robfon had had a tedious labour; was delivered, about fix that morning, of a healthy male child, rather fmall, and, as the abdomen was tenfe, fhe thought, perhaps, there might be another; the after-birth fhe could not deliver: fuppofing it adhered to the womb, the midwife and attendants had determined to truft to Nature. The patient had flooded conftantly from her delivery until the time I arrived, which was about twenty minutes paft ten, A. M. and had loft more blood than any perfon I had ever feen. The pulfe was become weak and tremulous; the countenance ghaftly and pale; eyes heavy and glazed, and feemed as if they almoft had loft their motion; a cold clammy fweat pervaded the whole body; her voice was feeble, and fhe had fainted twice. Applying my hand externally, I could eafily perceive there was not another child; this piece of information affifted in part to raife the drooping fpirits of the patient, who then fpoke for the firft time in my prefence; faid, fhe wifhed to be delivered of the after-birth. I carefully placed her in a proper pofition to attempt the extraction—I took hold of the funis, and, by pulling it very flightly, I immediately conceived a morbid adhefion had taken place; I then introduced my hand with all the gentlenefs and dexterity in my power, and found the uterus exceedingly contracted in the middle, like an hour-glass, which not unfrequently takes place; however, by cautiously and gradually dilating with one finger after the other, until I was able to pass my hand in a conical form, I then found two very large balls of coagulated blood, which I removed; I then felt for the placenta, which with difficulty I found adhering to the *fundus uteri*, almost as closely as if it had been a part of the uterus; from which, with perseverance, trouble and difficulty, I at last succeeded in separating it.

The hæmorrhage immediately ceased; my patient did not lose much blood during the operation; she complained of some slight pain, which was soon removed by a draught composed of the tinct. opii, &c. I ordered the admission and constant *supply* of fresh air; the bed, &c. to be kept particularly clean. As she was in a state of extreme debility, in consequence of the long continued and excessive hæmorrhage, I advised the use of tonics, astringents, and cordials.

I am happy to say she never had one bad symptom, but recovered wonderfully well, almost as much so as if nothing particular had taken place.

I am sorry to add, that R. Robson, about eighteen months after, died in child-bed. I was not desired to attend, of course I am perfectly unacquainted with the mode of treatment.

I must beg leave to make an observation or two upon the extraction of the placenta. When I first returned from Edinburgh, I was a great admirer of the spontaneous separation; I was taught to believe, that it was the best and most natural treatment to leave it almost always to Nature. If I had, in this case, deferred the operation for twelve or fourteen hours, and had trusted to Nature, what must have been the consequence? If art had not kindly interposed, Nature soon must have been exhausted, and death closed the scene.

Dr. Scott, who has had considerable practice, and who has been particularly successful, informed me, that he had met with several cases of morbid adhesion; and that, in about twelve cases, the extraction in all, except one, proved successful.

It has been the custom, in this part of Northumberland, in common cases, to deliver the placenta in about twenty minutes, or half an hour, after the delivery of the child, and this practice is perhaps as good as any that can be recommended; so strong is this prejudice here, that if you refuse to act, they immediately call in some other person.

If I was to hazard an opinion upon this very intersting subject, I would say, that when there is no flooding, it is proper to wait the time above mentioned, or a still longer. Where there there is any flooding of consequence after the delivery of the child, if it did not cease in a few minutes, after using proper remedies, I would then, without any hesitation, deliver the placenta.

At first sight this may appear a rude kind of practice; for some years I did not admire it; but it has been with us attended with the greatest success, for I know two or three cases where women have lost their lives by waiting too long for a spontaneous separation.

In Mr. White's, of Manchester, Treatise on the Management of Lying-in Women, I observe three cases, (of which he had been informed) where the women suffered by the placenta remaining, not from adhesion, but trusting to the efforts of nature. Vid. P. 306. Cases xiii. xiv. xv.

From these, and innumerable other cases, we ought to draw this invariable rule of practice, viz. *Never to quit a patient until the secundines are delivered.*

